# Association Between Family Structure and Anxiety Disorder Among Pre-schoolers: A Cross-Sectional Study in Urban Chongqing, China

**DOI:** 10.3389/fpsyt.2021.626377

**Published:** 2021-10-13

**Authors:** Hongmei Hu, Tingting Wu, Shanshan Wang, Peiling Chen, Jiaqiong Zhang, Xin Zhao

**Affiliations:** ^1^College of Pre-school Education, Chongqing University of Education, Chongqing, China; ^2^Children's Research Institute, Chongqing University of Education, Chongqing, China; ^3^Chongqing Collaborative Innovation Center for Functional Food, Chongqing University of Education, Chongqing, China; ^4^Family Education Guidance Center for 0-6 Years Old, Chongqing University of Education, Chongqing, China; ^5^Department of Food and Nutrition, College of Medical and Life Sciences, Silla University, Busan, South Korea; ^6^School of Public Health and Management, Chongqing Medical University, Chongqing, China

**Keywords:** pre-school anxiety scale (PAS), anxiety disorder, family structure, pre-schoolers, inter-generational family

## Abstract

**Objective:** This study explores the current situation of anxiety disorder of pre-schoolers and assesses the association between family structure and anxiety disorder (AD) among pre-schoolers in Chongqing, China.

**Methods:** This is a cross-sectional study of 499 main fosterers of children aged 3–6 years who completed the 28-item Chinese version of the Spence Pre-school Anxiety Scale (PAS). Multinomial logistic regression with three models was used to assess the association of the family structure with the different AD.

**Results:** The prevalence of AD was 31.46%, whose score of PAS were more than 48. Among the five different PAS sub-scales, the prevalence of obsessive-compulsive disorder (OCD) was the highest (50.10%), followed by separation anxiety disorder (SAD, 39.28%), fear of physical harm (FPH, 37.68%), generalized anxiety disorder (GAD, 33.47%), and social phobia (SP, 25.85%). Pre-schoolers from inter-generational families were more probably have AD than those from nuclear families (OR = 3.73, *p* < 0.05). The participants from inter-generational families were more likely to have SAD (OR = 3.39, *p* < 0.05), FPM (OR = 2.80, *p* < 0.05), or OCD (OR = 2.40, *p* < 0.05), in comparison with participants from other family structures.

**Conclusion:** Anxiety disorder among pre-schoolers aged 3–6 in Chongqing is widespread. Pre-schoolers from inter-generational families were more probably have AD, SAD, FPM, and OR and pre-schoolers from stem families may be less likely to have SAD compared with those from nuclear families. Relieving the anxiety of pre-schoolers may be possible with additional interventional efforts in inter-generational families.

## Introduction

Psychiatric disorders in pre-school children are becoming a public health problem, although their exact scope is not well-understood (1, 2). A general level of anxiety in a child can be regarded as a normal response, but if a child is excessively anxious, being in a state of high anxiety for a long time may lead to an overload of the nervous system and cause a series of psychological problems. Compared with adults, the emotion, cognition level, and physiological state of pre-schoolers are developing and changing. Their anxiety and terror are mainly expressed through behaviors, such as crying, tantrums, disobedience, or withdrawal behavior in response to unpleasant or difficult things, and they may have symptoms such as an accelerated heartbeat, poor sleep quality, or frequent urination (3). A state of high anxiety that continues for a long time may lead to excessive anxiety reactions, anxiety neurosis, or mental disorders, and may also increase the risk of depression in a child (4, 5). Anxiety disorders are widely acknowledged as the most prevalent class of psychiatric illness during the pre-school period and across the lifespan. Worldwide, the estimated prevalence of pre-school anxiety disorders by most studies in the range of 10–20% in recent decade (6, 7), even others report prevalence above 20% (8). Currently, in rapidly urbanized areas in China, the prevalence of anxiety disorders in pre-school children is as high as 34.2% (9). And retrospective studies report the median age of onset for anxiety disorders around 6 years of age (10, 11).

Nevertheless, factors and mechanisms contributing to pre-school-onset anxiety remain understudied. Existing research in determining the risk of pre-school anxiety disorder suggests strong influences on genetics, biological influences, gender, cognition, and environment (12). First, family history of internalizing difficulties, including high anxiety or depression, is a risk factor for pre-schoolers' anxiety disorders through genetic transmission (11, 13) or other mechanisms, like birth factor (14, 15) and breastfeeding (16). Temperament is one of the most powerful recognized variables in likelihood of acquiring anxiety disorder over life. Emotional, attention-like and motor reaction variations leading to new stimuli (17). Furthermore, behavioral inhibition was also consistently identified as a strong risk factor for pre-school development of anxiety disorder, and was linked to risk for pre-choice exuberance (11), low sociability, and low effortful control (13) have also been associated with risk for pre-school anxiety disorders (18).

Furthermore, levels of anxiety are decreasing gradually in pre-school children with age (19). Several other family-based factors have also been associated with risk for pre-school anxiety disorders that may operate through a combination of genetic and environmental influences. Parents who are younger, poorer, and less educated are more likely to have children with pre-school anxiety disorders (13, 20). The family function is also related to risk, as children who do not live with both biological parents and children with more siblings in the household are more likely to develop pre-school anxiety disorders relative to peers (6). If anxiety disorders of early childhood cannot be treated with timely intervention, they will continue into adolescence and even adulthood (21). Anxieties are often hidden, and they are often ignored by parents and teachers, which results in an increased likelihood that they will become aggravated and worsen (22).

Rapid urbanization, industrialization, and the aging of the population have occurred in China. With a population policy that has changed in recent years, the rates of cohabitation, divorce, and separation have become high in China, and this has led to noticeable changes in family structure and relationships in China (23). Extended families and various forms of Chinese families now exist, i.e., nuclear families, single-parent families, families with double income and no kid (DINKs), single-person households, and cohabitant households (24). With the one-child policy that was implemented for more than 20 years, there has been a significant increase in nuclear families of couples, and the growth rate of intergenerational lineal families is the highest (25). These social processes may have given rise to increasing heterogeneity in the types of families that children live in while growing up, and to increasing instability in family composition.

Family structure is a key factor that affects the mental health of children and adolescents (26). Children who live with both parents in a shared residence arrangement tend to have more optimal mental health outcomes than children who live with only one of their biological parents (27). Additionally, single-parent status is a risk factor for mental disorders in childhood and adolescence (28, 29). Compared to children who continuously live with both biological parents, children who live in one-parent, blended, or stepfamilies, as well as children who experience parental separation or divorce are more likely to suffer mental disorders (30). And different family structures with different parent-child relationships, the parents have different perceptions of children's anxiety disorder and play an important role in the development of anxiety selective mutism and generalized anxiety disorder (31, 32). As previous studies have pointed that pre-schoolers with high conflict in the home are more likely to experience significant anxiety relative to pre-schoolers in low-conflict homes (13).

However, there were few studies on family structure and anxiety in pre-schoolers in China, although there have been many studies on family education style (33), educational level of parents (34), and parent-child dependency status (19). The present study explores the current situation of anxiety among pre-schoolers and investigates the impact of different family structures on the anxiety level of pre-schoolers in Chongqing, China. By conceptualizing different family structures through family members who mainly live together and the main caregivers of pre-school children. We hypothesized that: (1) Sound family structure bears a negative relationship to anxiety level; and (2) the pre-schoolers from single-family and inter-generational families are more probably have higher anxiety.

## Materials and Methods

### Survey Procedure and Participants

A cross-sectional study was conducted. Five of the eight urban districts in Chongqing: Jiangbei District, Shapingba District, Nan'an District, Banan District, Wang Sheng area, were selected based on a convenient sampling method. For each district, two kindergartens were selected. The researcher explained the project details and do training on data collection quality control to the teachers of selected kindergartens before the survey.

Considering that pre-schoolers cannot efficiently answer the questionnaire, the main fosterer of the pre-schoolers was chosen to complete the survey. And the actual investigator was the pre-school teachers, who asked the parents to complete the questionnaire and answer questions if they volunteered participants in this project during parents' meetings held at the kindergartens in December 2019. Two months before the formal survey, we conducted a pre-survey and collected ~110 samples, and revised the questionnaire based on these results. All participating parents signed the informed consent forms before the formal survey.

A total of 560 questionnaires were sent out, and 499 valid questionnaires were recovered, with a response rate of 89.10%. The main fosterer included parents or grandparents of pre-schoolers or other family members who live with the child. The inclusion criteria of this study were as follows: (1) Children aged 3–6 years old who have not attended primary school, which based on the Chinese education law that makes it mandatory for every child to start attending primary school at age 6 or 7 years old (35); (2) The fosterer volunteered to participate in the survey and gave his or her informed consent; (3) The fosterer has a clear understanding of children, which is the children's information provided by the main fosterer is consistent with the information that provided at the time of admission.

### Measures

#### Measurements of Anxiety of Pre-schoolers

The 28-item Chinese version of the Spence Pre-school Anxiety Scale (PAS) was used to evaluate the anxiety status of pre-school children in this study. The Chinese version of the Spence Pre-school Anxiety Scale (PAS) was specifically designed by Chinese researchers to be used with pre-schoolers aged 3–6 years in China with the Cronbach's α coefficient is 0.95 (34). The questionnaire consists of five subscales: separation anxiety disorder (SAD), fear of physical harm (FPH), social phobia (SP), obsessive-compulsive disorder (OCD), and generalized anxiety disorder (GAD) to measure these five types of anxiety and post-traumatic stress disorder. A five-point scoring method is used to indicate the anxiety disorder for each item, and the score is a range from 0 to 4 (0 = never; 1 = rarely; 2 = sometimes; 3 = often; 4 = always). The sum of the original scores of each item is used as the total score of PAS. If the total score is ≥48 points, which was defended as an anxiety disorder (AD). The higher the score for each different PAS sub-scale, the more severe the child's anxiety. Considering the lack of clear criteria for the PAS sub-scale in China, we used the demarcation point of anxiety from previous Chinese studies as a reference (>2SD) (34). A score of separation anxiety subscale >7 is defined as SAD, and <7 is divided into the normal group. A score of fear of physical harm subscale >12 is defined as FHP, or it is divided into the normal group. The score of social phobia subscale >11 is defined as SP, and a score of obsessive-compulsive disorder subscale >5 is defined as OCD, and a score of generalized anxiety subscale >7 is defined as GAD.

#### Independent Variables

The family structure was divided into five categories based on previous literature: (1) Nuclear family (36): a family group that consists only of parents and children. (2) Single-parent family (29): a single parent individual who shoulders most or all of the day-to-day responsibilities for raising a child or children, due to death of the partner, divorce, or unplanned pregnancy. (3) Blended family (24): the family form created in a remarriage that involves one or more children from the previous marriage of either spouse. (4) Stem family: children living with Grandparents and parents but where the parents have primary care responsibilities. (5) Inter-generational family (24): a family where the children only living with grandparents and the parents are not present.

#### Covariates

The age of the child, birth weight, breastfeeding duration, premature infant status, family structure, educational level of the father, educational level of the mother, and per capita annual family income were described as the socioeconomic status information of pre-schoolers. These data were employed as covariates, as these variables were discussed in previous studies (9, 15, 30). Age and birthweight were treated as continuous variables, and all other variables were treated as categorical variables. Breastfeeding duration was categorized into three levels (<6 months, more than 6 months but no more than 12 months, or more than 12 months). Premature infant status was divided into yes or no. Education level and per capita annual family income were both categorized into three levels (low, medium, and high).

#### Statistical Analysis

Descriptive statistics were used to show the basic information of pre-schoolers with different socio-demographic statuses. The chi-square test was used for categorical variables and ANOVA for continuous variables. The prevalence of AD was measured by estimated mean values. Comparison analyses of AD prevalence between different family structures were conducted by chi-square analysis. Gender, age, birthweight (14), premature birth (15), and breastfeeding duration (16, 37), and family income were identified to determine how they affected the pre-schoolers' anxiety. Logistic regression was performed to assess the association of family structure with AD using three models. Model 1 was adjusted for sex, age, birth weight, premature birth, and breastfeeding duration. Model 2 was adjusted to be the same as Model 1 plus the number of children, fathers' education level, mothers' education level, and per capita household income. Model 3 was adjusted to be the same as Model 2 plus Model 1. The regression models were fully adjusted for the same set of control variables. According to the criteria for assessing model fit, the log-likelihood statistics of the fitted model indicate a satisfactory fit of the model. The results are reported for the odds ratio (OR) and 95% confidence interval (95% CI). All these analyses were performed using Stata version 15.0 software (Stata Corporation, College Station, TX, USA).

## Results

### Characteristics of the Study Sample

Table 1 presents the descriptive demographic variables for the study sample. Nearly half of the pre-schoolers were boys (51.10%). The average age of the pre-schoolers in this study is 5.02 years old, and most of the pre-schoolers were not premature infants (88.18%). More than 50% of the pre-schoolers were breastfed for more than 6 months, but no more than 12 months. The educational level of the pre-schoolers' parents was not low, with nearly 50% receiving higher education. However, 48.10% of the participants were from low per capita household income families. We found that the number of pre-schoolers who came from nuclear families (44.09%) was larger than that of other family structures. The percentages of pre-schoolers from a stem family, single-parent family, and inter-generational family were 26.85, 14.43, and 8.82%, respectively.

**Table 1:** Demographic data of pre-schoolers in the study (*N* = 499).

**Demographics**	**Groups**	***N* (n%)/mean ± SD**
Gender	Boy	255 (51.10%)
	Girl	244 (48.90%)
Age		5.02 ± 1.02
Birthweight		3.21 ± 0.43
Pre-mature	No	440 (88.18%)
	Yes	59 (11.82%)
Time of breastfeeding	<6 months	176 (35.27%)
	more than 6 months but no more than 12 months	255 (51.10%)
	more than 12 months	68 (13.63%)
Education level of father	Low	171 (34.27%)
	Medium	86 (17.23%)
	High	242 (48.50%)
Education level of mother	Low	181 (36.27%)
	Medium	87 (17.43%)
	High	231 (46.29%)
Income	Low	240 (48.10%)
	Medium	158 (31.66%)
	High	101 (20.24%)
Structure of family	Nuclear family	220 (44.09%)
	Stem family	134 (26.85%)
	Single-parent family	72 (14.43%)
	Inter-generational family	44 (8.82%)
	Blended family	29 (5.81%)

*Data are presented as Mean ± SD for continuous measures, and n (%) for categorical measures*.

### Current Situation of Anxiety Status of Pre-schoolers With Different Family Structures

There were 157 children whose score on the PAS scale was more than 48, which showed that the prevalence of total anxiety disorder was 31.46% in this study. Among the five different PAS sub-scales (Table 2), the prevalence of OCD was the highest (50.10%), followed by SAD (39.28%), FPH (37.68%), and GAD (33.47%), and the prevalence of SP was the lowest (25.85%). The prevalence of AD in pre-schoolers from intergenerational families was the highest (61.36%), and the prevalence of AD in pre-schoolers from nuclear families was the lowest (24.9%). As for the prevalence of SAD (68.18%), FPM (61.36%), OCD (68.18%), GAD (59.09%), SP (52.27%) in pre-schoolers from intergenerational families were most the highest compared to other family structures. Significant differences were found between family structures and the prevalence of anxiety disorders (*p* < 0.001), while OCD was not significantly different among the different family structures (*p* > 0.05).

**Table 2:** Prevalence of d Anxiety disorder and disorder of the five different PAS sub-scales by family structure.

	**Total**	**Nuclear family**	**Single-parent family**	**Blended family**	**Stem family**	**Inter-generational family**	***p*-value**
	**(*N* = 499)**	**(*n* = 220)**	**(*n* = 72)**	**(*n* = 29)**	**(*n* = 134)**	**(*n* =4 4)**	
Anxiety disorder	157 (31.46%)	53(24.09%)	24 (33.33%)	11 (37.93%)	42 (31.34%)	27 (61.36%)	<0.001^**^
**The five different PAS sub-scales**							
SAD	196 (39.28%)	75 (34.09%)	29 (40.28%)	15 (51.72%)	47 (35.07%)	30 (68.18%)	<0.001^**^
FPM	188 (37.68%)	71 (32.27%)	25 (34.72%)	13 (44.83%)	52 (38.81%)	27 (61.36%)	0.007^*^
SP	129 (25.85%)	45 (20.45%)	16 (22.22%)	7 (24.14%)	38 (28.36%)	23 (52.27%)	<0.001^**^
OCD	250 (50.10%)	100 (45.45%)	39 (54.17%)	15 (51.72%)	66 (49.25%)	30 (68.18%)	0.085
GAD	167 (33.47%)	49 (22.27%)	29(40.28%)	14 (48.28%)	49 (36.57%)	26(59.09%)	<0.001^**^

* *p <0.05*;

** *p <0.01. The five subscales: separation anxiety disorder (SAD), fear of physical harm (FPH), social phobia (SP), obsessive-compulsive disorder (OCD), and generalized anxiety disorder (GAD)*.

### Association Between Family Structure and Anxiety Score of Pre-schoolers

Table 3 shows a multivariable-adjusted association between different disorders and different family structures in fully adjusted models (model 3). Compared with nuclear families, pre-schoolers from inter-generational families were more likely to have AD (OR = 3.73, 95% CI = 1.76, 7.89). participants from inter-generational families were more likely to have SAD (OR = 3.39, 95% CI = 1.63, 7.04), FPM (OR = 2.80, 95%CI = 1.39, 5.65), or OCD (OR = 2.40, 95% CI = 1.16, 5.04) than those from other family structures. The participants from nuclear families were less likely to have GAD compared with pre-schoolers from the other family structures (*p* < 0.05). Additionally, we found that participants from stem families were less likely to have SAD (OR = 0.85, 95% CI = 0.53, 1.38), but not significantly (*p* > 0.05).

**Table 3:** Association (OR) between family structure and different disorders by multivariable logistics regression analysis regression among preschoolers.

**Family structure**	**AD**	**SAD**	**FPM**	**SP**	**OCD**	**GAD**
	**OR (95%CI)**	**OR (95%CI)**	**OR (95%CI)**	**OR (95%CI)**	**OR (95%CI)**	**OR (95%CI)**
Nuclear family	Ref.	Ref.	Ref.	Ref.	Ref.	Ref.
Single-parent family	1.41 (0.75–2.66)	1.23 (0.69–2.20)	1.11 (0.62–2.00)	0.99 (0.49–1.98)	1.34 (0.76–2.35)	2.37^*^ (1.29–4.35)
Blended family	1.90 (0.79–4.58)	2.05 (0.91–4.60)	1.73 (0.77–3.88)	1.31 (0.50–3.45)	1.27 (0.57–2.83)	3.73^*^ (1.61–8.65)
Stem family	1.02 (0.61–1.72)	0.85 (0.53–1.38)	1.18 (0.73–1.88)	1.19 (0.70–2.02)	1.00 (0.63–1.58)	1.68^*^ (1.02–2.78)
Inter-generational family	3.73^*^ (1.76–7.89)	3.39^*^ (1.63–7.04)	2.80^*^ (1.39–5.65)	0.99 (0.49–1.98)	2.42^*^ (1.16–5.04)	4.38^*^ (2.11–9.07)

* *p <0.05; The Nuclear family was treated as the reference category. The gender, age, birth weight, premature and time of breast, number of children, fathers' education level, mothers'education level, and per capita household income were adjusted. The anxiety disorder (AD) and five subscales: separation anxiety disorder (SAD), fear of physical harm (FPH), social phobia (SP), obsessive-compulsive disorder (OCD), and generalized anxiety disorder (GAD)*.

## Discussion

In the current study, we have assessed whether and how family structure is associated with the prevalence of childhood anxiety disorder among children aged 3–6 years in Chongqing, China. The results showed that the prevalence of anxiety disorder among pre-schoolers in Chongqing, China was high, especially the prevalence of OCD and SAD. This finding was higher than the data from the USA (6, 38, 39) and Europe (40, 41). The norms and obedience emphasized in Chinese culture may cause high anxiety disorder in children. These findings were similar to those for pre-schoolers in rapidly urbanized areas (9). The urbanization rate of Chongqing is increasing year by year, and it was 65.5% in 2018 (42), which may be one of the reasons for the higher prevalence of anxiety. Children growing up in the context of rapid urbanization experienced dramatic changes in their environment in the context of social change, instability, environmental changes, increased competition, and studies of left-behind and migrant children resulting from urbanization have demonstrated the significant impact of social change on the mental health of children and adolescents (43). This is similar to previous research findings in China, where the rapidly changing and evolving social environment during transition and urbanization can cause widespread anxiety and fear in children who are new to the world (44).

What is different from previous studies (45, 46) is that the prevalence of OCD was the highest, followed by SAD, and the prevalence of SP was the lowest in this study. The average age of the participants was 5 years old in this study, which may have caused the difference because previous indications are that older pre-schoolers are more likely to have OCD (12). The prevalence of anxiety in pre-schoolers from nuclear families was the lowest, which is in accordance with the results of a previous study that indicated that children in original families are approximately half as likely as children in step, blended, or one-parent families to experience any mental disorder (26). AD of pre-schoolers from different family structures in Chongqing China is widespread, and timely intervention is necessary.

There were substantial and statistically significant differences in the prevalence of AD among pre-schoolers who live in different types of families. The family modernization of China followed in the footsteps of that in Western industrial societies. The rates of cohabitation, divorce, and separation have become high in China (23). These social processes may have given rise to increasing heterogeneity in the types of families in which children live while growing up and also to increasing instability in family composition. We found that the possibility of anxiety disorders including SAD, FPM, SP, and GAD was not statistically different for pre-schoolers in single-parent families, pre-schoolers in blended families, and children in stem families compared with those from nuclear families. These results did not support the usual social narratives arguing that children who live in one-parent, blended, and stepfamilies, as well as children who experience parental separation or divorce, carry the highest burden of morbidity (28, 29), which was consistent with prior evidence indicating that children in traditional families experience more positive socio-emotional outcomes than children in other family types in Australia (26). The reason for these results may be that even in single-parent families, the care given by their biological parents has not diminished. It is very common to parent children together after divorce in China.

We found that the possibility of anxiety disorder including SAD, FPM, OCD, and GAD in pre-schoolers from inter-generational families was higher than those from nuclear families in a multivariate model that adjusted for gender, age, birth weight, premature birth, breastfeeding duration, and family situation (number of children in the family, fathers' education level, mothers' education level, and per capita household income). This finding was similar to that of a previous study performed in Anhui, which revealed that children currently left behind by their migrating parents demonstrated greater susceptibility to mental health difficulties than urban children living with their parents (47). There are an estimated 61 million city-to-city migrants in China, accounting for roughly 25% of the total migrant population, and it is increasing (48). The increase in this type of migration has resulted in a large and ever-growing population of urban inter-generational families that consist of only grandparents and grandchildren, which accounted for 30.79% (47, 48).

Pre-schoolers living with their grandparents usually have little communication with their parents, and the lack of parent-child interaction results in a lack of an attachment relationship that reduces their sense of safety in pre-schoolers. This psychological shock results in children that are not at ease and can easily panic, leading to a high level of separation anxiety (49). However, grandparents' love for their grandchildren is not restricted, as grandparents are very fond of their grandchildren in traditional Chinese culture. This is still the case today, especially in inter-generational families where parents do not live with their children due to work or other reasons. In inter-generational families, grandparents often overprotect their grandchildren to avoid risks. They inform grandchildren of dangers very frequently, which can easily lead to FPH. At the same time, they may reduce grandchildren's opportunities for outdoor activities, which affects the physical activities and social skills of pre-schoolers, thereby increasing the possibility of grandchildren suffering from anxiety. Inter-generational families are not unique to China. In other countries around the world, intergenerational families are mainly formed due to parents' drug abuse or death (50). The current study shows that intergenerational families living alone with grandparents may not be conducive to the development of pre-school children's mental health.

The findings of this study may be of help to design tailored prevention programs among high-risk families, which would allow us to adequately intervene over potentially modifiable variables. In the current study, it could be argued that the family structure is variable in the evaluation of pre-schoolers in psychiatric settings, and further research in this regard is warranted, which may fill the gaps in the current literature and contribute to anxiety disorder intervention for different types of family structures in China. However, the results should be interpreted with caution. Firstly, this cross-sectional study cannot be taken as evidence that family structure produces children's anxiety disorders or vice versa. In this regard, some authors have argued that formal aspects of family structure may be mediators of the effect of other variables, such as family rearing or parenting styles, on the development of psychopathology (51). Given the nature of our dataset, we were unable to evaluate these aspects in our study, but we adjusted the educational level of parents, the number of children in the family, and household income. This is an area that deserves further investigation. Secondly, parent-reported which may cause high or low anxiety disorder might be a challenge to this study and this reporting is subject to dishonest responses or acquiescence bias, although it is a common limitation for most survey-based research. Furthermore, the study of risk factors for the development of childhood anxiety disorders is crucial. Additionally, the higher self-reported prevalence of AD of children from intergenerational families may be caused by the reporter of intergenerational families may be the grand-parent. The age of the reporter may influence in reporting about the anxiety of the child. This may be a confounding factor, which needs further research.

## Conclusion

Anxiety Disorder among pre-schoolers aged 3–6 in Chongqing, China is widespread. Obsessive-compulsive disorder was the most prevalent, followed by separation anxiety and fear of physical harm. Pre-schoolers from intergenerational families were more likely to have Anxiety Disorder, including SAD, FPH, SP, OCD, or GAD than other family structures. In contrast, pre-schoolers from stem families may be less likely to have separation anxiety disorder compared with pre-schoolers from nuclear families. Inter-generational families may be a high-risk factor for anxiety disorder. Decreasing the anxiety of pre-schoolers may be possible with more interventional efforts in inter-generational families. In addition to pre-schoolers from nuclear families, the root cause of anxiety among children from other family structures is the lack of adequate parental care. To alleviate the anxiety disorder of children, parents should communicate with their children more frequently and concern about them.

## Data Availability Statement

The original contributions presented in the study are included in the article/supplementary material, further inquiries can be directed to the corresponding author/s.

## Ethics Statement

This study was approved by Ethics Committee Review Committee of Chongqing Collaborative Innovation Center for Functional Food in Chongqing University of Education (201901HS02). This study was conducted in accordance with the Declaration of Helsinki. The teachers provided informed consent before participating in the study. Written informed consent to participate in this study was provided by the participants' legal guardian/next of kin.

## Author Contributions

HH and TW conducted statistical analyses of the data and prepared the draft manuscript. SW edited the manuscript. PC contributed to the data curation. XZ and JZ provide critical comments to the manuscript. All authors checked and proofread the final version of the manuscript.

## Funding

This research was funded by Chongqing University Innovation Research Group Project (CXQTP20033), Humanities and Social Sciences Research Project of Chongqing Municipal Education Commission (21SKGH278), Children's Research Institute of National Center for Schooling Development Program and Chongqing University of Education (CSDP19FS01103), and the National Education Science Project Youth Program of the Ministry of Education Research on the Upgrading Mechanism for Public Service Capacity of Southwestern Rural Preschool Education from the Perspective of Rural Revitalization Strategy (EHA180483), China.

## Conflict of Interest

The authors declare that the research was conducted in the absence of any commercial or financial relationships that could be construed as a potential conflict of interest.

## Publisher's Note

All claims expressed in this article are solely those of the authors and do not necessarily represent those of their affiliated organizations, or those of the publisher, the editors and the reviewers. Any product that may be evaluated in this article, or claim that may be made by its manufacturer, is not guaranteed or endorsed by the publisher.
